# Copper(II)-Doped Carbon Dots as Catalyst for Ozone Degradation of Textile Dyes

**DOI:** 10.3390/nano12071211

**Published:** 2022-04-04

**Authors:** Rita M. F. Cardoso, Inês M. F. Cardoso, Luís Pinto da Silva, Joaquim C. G. Esteves da Silva

**Affiliations:** Chemistry Research Unit (CIQUP), Institute of Molecular Sciences (IMS)—DGAOT, Faculty of Sciences of University of Porto (FCUP), Rua do Campo Alegre 697, 4169-007 Porto, Portugal; up201704723@edu.fc.up.pt (R.M.F.C.); up201704720@edu.fc.up.pt (I.M.F.C.); luis.silva@fc.up.pt (L.P.d.S.)

**Keywords:** textile dyes, ozone, nanomaterials, AOPs, wastewater treatment, carbon dots

## Abstract

A catalytic ozonation advanced oxidation process (AOP) with a copper(II)-doped carbon dot as catalyst, Cu-CD (using L-cysteine and polyethylene glycol (PEG) as precursors and passivation agents), was developed for textile wastewater treatment (T = 25 °C and pH = 7). Four dyes were analyzed—Methyl Orange (MO), Orange II sodium salt (O-II), Reactive Black 5 (RB-5) and Remazol Brilliant Blue R (RBB-R), as well as a real effluent from the dying and printing industry. The Cu-CD, with marked catalytic ozonation properties, was successfully synthesized by one-pot hydrothermal procedure with a size of 4.0 nm, a charge of −3.7 mV and a fluorescent quantum yield of 31%. The discoloration of the aqueous dye solutions followed an apparent first-order kinetics with the following rate constants (*k*_ap_ in min^−1^): MO, 0.210; O-II, 0.133; RB-5, 0.177; RBB-R, 0.086. In the presence of Cu-CD, the following apparent first-order rate constants were obtained (*k*_ap_^c^ in min^−1^) with the corresponding increase in the rate constant without catalyst (%Inc): MO, 1.184 (464%); O-II, 1.002 (653%); RB-5, 0.709 (301%); RBB-R, 0.230 (167%). The presence of sodium chloride (at a concentration of 50 g/L) resulted in a marked increase of the discoloration rate of the dye solution due to generation of other radicals, such as chlorine and chlorine oxide, resulting from the reaction of ozone and chloride. Taking into consideration that the real textile effluent under research has a high carbonate concentration (>356 mg/L), which inhibits ozone decomposition, the discoloration first-order rate constants without and with Cu-CD (*k*_ap_ = 0.0097 min^−1^ and *k*_ap_^c^ = 0.012 min^−1^ (%Inc = 24%), respectively) were relatively small. Apparently, the Cu-CD, the surface of which is covered by a soft and highly hydrated caramelized PEG coating, accelerates the ozone decomposition and dye adsorption, increasing its degradation.

## 1. Introduction

The maintenance of the quality of environmental water is a mandatory objective to achieve sustainable development [1]. Industrial sewers are major sources of water contamination, with textiles being an example of an activity causing water pollution, particularly with dye substances [2]. To eliminate dyes and their subproducts from textile effluents, advanced oxidation processes (AOP) are usually required [3]. Ozone is a highly versatile AOP that can be easily implemented in pre-existing wastewater treatment facilities [4].

By bubbling ozone gas in water, it is transferred into an unstable aqueous solution with a short half life that may be in the seconds time range [5]. In the decay of ozone, the major secondary oxidant form is the hydroxyl radical (*OH). The decomposition of ozone initiates with the reaction with hydroxide ions, involving reactions (1) to (3); depending on the pH of the aqueous solution, it evolves according to the different mechanisms depicted in reactions (4) to (8) [5].

O_3_ + OH^−^ → HO_2_^−^ + O_2_ (1)

O_3_ + HO_2_^−^ → *OH + O_2_*^−^ + O_2_ (2)

O_3_ + O_2_*^−^ → O_3_*^−^ + O_2_ pH <≈ 8(3)

O_3_*^−^ + H^+^ ⇌ HO_3_* (4)

HO_3_*^−^ → *OH + O_2_ pH >≈ 8(5)

O_3_*^−^ ⇌ O*^−^ + O_2_ (6)

O*^−^ + H_2_O **→** *OH + OH^−^ (7)

*OH + O_3_ → HO_2_* + O_2_ (8)

The oxidation potential of an ozone solution and, consequently, its reactivity in dye degradation, corresponds to the sum of the concentration of ozone and hydroxide radicals. The propagation of the ozone decomposition chain reaction provokes a rapid decrease in the aqueous ozone concentration, which can only be terminated by inhibitors, such as natural organic matter and carbonates [5]. The hydroxyl radical is the most active oxidant in the dye degradation processes; consequently, with a continuous source of ozone, the existence of factors that destabilizes ozone, i.e., catalysts, increase the degradation efficiency of the process.

Ozone is a common AOP of textile wastewater [6,7]. Ozone can degrade a wide range of textile dyes, promoting their discoloration and biodegradability. Indeed, ozone and/or the hydroxyl radical tend to break multiple chemical bounds that are responsible for the color of dyes [8]. Additionally, ozone increases the biodegradability of textile wastewater by breaking chemical bounds of refractory organic compounds, originating low-molecular-weight, easily biodegraded molecular fragments [9]. Recently, catalysts have been coupled to ozone (catalytic ozonation) to increase efficiency making the ozone a low selective AOP for pollutant removal in aquatic environments [6].

Nanomaterials have been coupled with ozone (catalytic ozonation) to eliminate or reduce the number of chemical substances involved in these treatments while increasing the pollutant degradation yields and reducing environmental impacts [10,11,12,13]. Taking into consideration natural resources, environmental sustainability and toxicity issues, carbon-based nanomaterials are preferred to metallic/semi-metallic materials in real-world applications [14]. Among such carbon-based nanomaterials, the synthetic versatility of carbon dots (CDs) [15,16,17,18,19,20,21,22,23,24] makes them the preferred choice for coupling to an AOP for the treatment of textile wastewater.

The synthesis of a CD is designed to obtain a desirable reactivity. In this paper, we describe the synthesis of a CD to catalyze the decomposition of ozone, generating more radical species, particularly the hydroxyl radical, and improving dye decomposition or wastewater discoloration. In this case, the rationale for the synthesis design had the following three criteria:

(i). To destabilize an oxidant such as ozone, promoting its decomposition, an antioxidant is necessary, and L-cysteine will be used as carbon precursor. Moreover, it has been observed that antioxidant activity is related to –SH functional groups on CD [25];
(ii). To increase the surface reactivity of the CD, doping with transition metal ions, that are rich in electrons, will provide a means for emerging chemical reactivities [26]. For example, copper(II) doping of CD can boost electron transfer and photooxidation [27] and has already been used in several applications [26,27,28,29];
(iii). To increase the water solubility and to coat the CD with a soft surface, allowing increased affinity of ozone and/or dye molecules, polyethylene glycol (PEG) will be used as a carbon precursor (together with L-cysteine) and as a passivating agent. Indeed, PEG undergoes caramelization, leading to the production of PEGylated nanoparticles [30]. Moreover, PEG is a highly water-soluble polymer and has the capability to enhance the CD quantum yield [31,32,33].

In this paper, we describe the synthesis of a copper(II)-doped carbon-based nanomaterial (carbon dot, Cu-CD) to be used as a homogeneous catalyst in the ozone discoloration of dyes present in the wastewater of textile industries: Methyl Orange (MO), Orange II sodium salt (O-II), Reactive Black 5 (RB-5) and Remazol Brilliant Blue R (also known as Reactive Blue 19) (RBB-R). However, the optimal preparation of the catalyst was subject to a preliminary exploratory synthesis analysis using different carbon antioxidant precursors (L-cysteine, citrate or ascorbic acid), three transition metal ions (copper(II), zinc(II) or titanium(IV)) and the analysis of the necessity of PEG in the former formulation. After this preliminary exploratory analysis and using the best-performing substances (L-cysteine, copper(II) and PEG), a Plackett–Burman experimental design was implemented to obtain the optimal synthesis conditions, i.e., those conditions that originate a nanomaterial (Cu-CD) with the highest catalytic ozonation discoloration of dyes. Moreover, for comparison purposes, another CD was synthesized without PEG, i.e., with only L-cysteine and copper(II), and it was named CuCys-CD.

To the best of our knowledge, CDs have never been designed as homogeneous catalysts in ozone AOP [7]. On the contrary, CDs are being incorporated in heterogeneous catalysts for the photodegradation of organic pollutants [34,35,36].

## 2. Materials and Methods

### 2.1. Reagents

**Figure 1:**
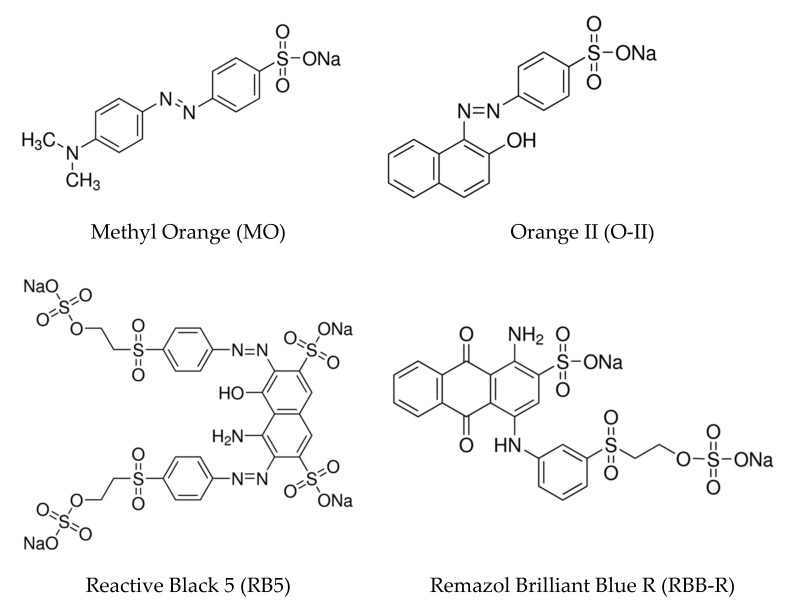
Chemical structures of the four dyes used in this work. MO, O-II and RB5 belong to the azo family of dyes, and RBB-R is an anthraquinone dye.

Polyethylene glycol with average molecular weight 200 (PEG) (Sigma-Aldrich, Burlington, MA, USA, P3015), copper(II) nitrate (Merck, Darmstadt, Germany, 0140630), L-cysteine (Sigma-Aldrich, W326305), ammonium citrate dibasic (Fluka Analytical, 09831), ascorbic acid (Merck, F971227), zinc nitrate (Merck, CC396733), titanium(IV) isopropoxide (Aldrich Chemistry, 87560), isopropyl alcohol (Merck, 1.09634) and sodium chloride (Fluka, S9888), reagents of analytical grade, were acquired from Sigma-Aldrich. The dye references were: Methyl Orange (Fluka, 68250), Orange II sodium salt (Aldrich, 195235), Reactive Black 5 (Sigma-Aldrich, 306452) and Remazol Brilliant Blue R (Sigma, R8001), acquired from Sigma-Aldrich. The chemical structures of the dyes are shown in Figure 1.

A typical real textile effluent was obtained from a textile dying and printing industry located in Barcelos (Portugal), with the following features: COD, 339 ± 9 mg O_2_/L; turbidimetry, 57.7 ± 0.3 NTU; pH, 8.66 ± 0.01; conductivity, 4.61 ± 0.01 mS/cm; chloride anion, 100 mg/L; carbonates, >356 mg/L. Moreover, optical microscopy analysis of the raw textile effluent showed the presence of a considerable number of textile fibers, microplastics and microorganisms (raw effluent is a mixture of industrial effluent and sanitary sewage).

### 2.2. Synthesis of Copper(II)-Doped Carbon Dots (Cu-CDs and CuCys-CDs)

A one-pot bottom-up hydrothermal process was used for Cu-CD synthesis. To obtain an optimal synthesis condition of the Cu-CD with higher ozonation catalytic features, a Plackett–Burman experimental design with two central experiments (a total of 10 experiments) was used. The following experimental factors were optimized: volume of PEG (1, 2 and 3 mL), mass of L-cysteine (0.1, 0.25 and 0.4 g in 15 mL of water), volume of the copper(II) solution (0.50, 0.75 and 1.0 mL of a copper(II) nitrate solution prepared by mixing 1.00 g in 1 mL of water), reaction temperature (180, 190 and 200 °C) and time of reaction (5.0, 7.5 and 10 h). The resulting solution was placed in a Parr Series 4700 pressure vessel and treated hydrothermally for the given timespan at the required temperature of the experiment. At the end of the reaction, the solid residual was separated by centrifugation, and the supernatant was dialyzed (Spectra-Por Float-A-LyzerG2, Rancho Dominguez, CA, USA; cutoff of 100–500 Daltons) for 24 h followed by lyophilization.

The optimal conditions (those originating the best ozone AOP catalyst) for the synthesis of Cu-CD were: volume of PEG, 3 mL; mass of L-cysteine, 0.1 g in 15 mL of water; volume of the copper(II) solution, 0.50 mL of a copper(II) solution prepared by mixing 1.00 g in 1 mL of water; reaction temperature, 180 °C; and time of reaction, 5.0 h. Another sample of carbon dots, named CuCys-CD, was synthesized using a similar procedure but without PEG.

### 2.3. Ozone Advanced Oxidation Process (AOP)

An ozone generator (A2Z OZONE) was employed to produce ozone from air that was bubbled at the bottom of a 1 L beaker by means of a diffuser at a mass flux of 47.5 mg/h and at a volumetric flux of 0.4 mL/min (titrated with potassium iodide and a standard solution of sodium thiosulfate [37]). All experiments were carried out at pH = 7.0 and at a temperature of 25 °C in batch mode on a sample volume of 1.00 L (images in SI). The pH of the aqueous solution to be ozonated was adjusted to 7.0 with sodium-hydroxide- and hydrochloric-acid-diluted solutions. At the end of the experiments, the pH of the treated solutions was measured and decreased to about 5.

The initial concentration of the dye aqueous solutions were: MO—2.06 × 10^−5^ mol/L (6.74 mg/L), O-II—1.84 × 10^−5^ mol/L (6.45 mg/L) and RB-5—6.75 × 10^−6^ mol/L (6.69 mg/L); RBB-R—1.75 × 10^−4^ mol/L (110 mg/L). For the analysis of the effect of the sodium chloride on the degradation, 50 g of NaCl was dissolved in the dye solution (50 g NaCl/L). For the analysis of the effect of the Cu-CD, 0.500 mL of the dialyzed solution was used.

In the experiments designed to confirm that the hydroxyl radical was involved in the catalysis mechanism, 0.800 mL of isopropyl alcohol, a hydroxyl scavenger, was added to the 1.00 L MO dye before ozonation.

### 2.4. Equipment

The morphology and chemical composition of the materials were obtained by scanning electron microscopy (Quanta 200 from FEI Company, Hillsboro, OR, USA) coupled with energy-dispersive spectroscopy (SEM-EDS). Fluorescence analysis was measured in a 10 mm fluorescence quartz cell using a Horiba Jobin Yvon FluoroMax spectrofluorimeter (Piscataway, NJ, USA) with 5 nm slit widths. AFM analysis was carried out using a Veeco (Plainview, NY, USA) metrology multimode/nanoscope IVA in tapping mode with a Bruker (Billerica, MD, USA) silicon probe (model, TESP-SS; resonant frequency, 320 kHz; nominal force constant, 42 N/m; estimated tip radius, 2 nm). Zeta potential was measured using an Anton Paar LitesizerTM 500 particle analyzer (Graz, Austria) and a polycarbonate omega cuvette (Ref. 155765) (Graz, Austria). The chemical states of the elements were determined by X-ray photoelectron spectroscopy (XPS) analysis using a Kratos AXIS Ultra HAS instrument (Kyoto, Japan). The analysis was performed with a monochromatic Al Kα X-ray source (1486.7 eV), and data were processed with CasaXPS software (Casa Software Ltd., Teignmouth, United Kingdom). Fourier transform infrared (FTIR) spectra were measured with a Perkin-Elmer (Waltham, MA, USA, EUA) Spectrum Two with an ATR sampling accessory. X-ray diffraction (XRD) patterns were collected using a high-resolution X-ray diffractometer (Rigaku SmartLab; X-ray source: copper (Cu); 9 kW rotating anode; Bragg–Brentano focusing method; K-Beta filter; detector: D/teX Ultra 250 silicon strip detector; 1D mode).

Textile wastewater was analyzed using the following equipment: COD: Hanna Instruments (Woonsocket, Rhode Island, United States) reagent set HI94754B-25, HI 839800 COD reactor and HI83214 multiparameter bench photometer for wastewater treatment application; turbidimetry: Hanna Instruments HI83200 multiparameter photometer (Woonsocket, RI, USA); pH: Fisher brand Accumet AB150 (Singapore); conductivity: Crison conductivity meter GLP 31 (Barcelona, Spain); chloride anion:Quantofix, Macherey-Nagel Ref. 91921 (Duren, Germany); carbonates: Quantofix, Macherey-Nagel Ref. 91323 (Duren, Germany).

### 2.5. Data Analysis

Monitoring of the absorbance of the dye aqueous solutions as function of the time of ozonation was carried out using a UV-Vis spectrophotometer (VWR, UV-3100PC spectrophotometer) at the maximum absorbance of the dye: MO—462 nm; O-II—485 nm; RB-5—598 nm; RBB-R—596 nm. The percentage of dye removal (%DR), the apparent pseudo-first-order rate constant (*k*_ap_ without catalyst and *k*_ap_^c^ with catalyst) and the percentage increase in the rate constant when the catalyst was present (%Inc) were calculated using Equations (9)–(11), respectively (a linear relation between the absorbance and the aqueous concentration of the dyes was observed):

%DR = 100 × (Abs_0_ − Abs_t_)/Abs_0_ (9)

ln(Abs_t_/Abs_0_)= − *k*_ap_ *t* (10)

%Inc = 100 × (*k*_ap_^c^ − *k*_ap_) / *k*_ap_ (11)

where Abs_0_ and Abs_t_ are the absorbances of the dye aqueous solution at initial and *t* time of ozonation.

The experimental design formulation and the analysis of the results (the apparent rate constant of the ozone degradation of MO in the presence of the synthesized Cu-CD) were conducted with Unscramble Design v9.6 (CAMO Software AS, Oslo, Norway).

Fluorescence quantum yield (QY) was calculated by the comparison of the integrated luminescence intensities and absorbance values of the Cu-CD with quinine sulfate (QY_QS_ = 0.54) using Equation (12):

QY = QY_QS_ × (Grad_Cu-CD_/Grad_QS_) × (η^2^/η^2^_QS_) (12)

where Grad is the gradient from the plot of integrated fluorescence intensity versus absorbance and η is the refractive index [38].

## 3. Results

### 3.1. Ozone AOP of Dyes

The ozone AOP of the aqueous solutions of the dyes resulted in an almost completed reduction of the color (Figure S1 in Supplemental information). The analysis of the spectra as a function of the reaction time shows that the visible part of the spectra decreases the absorbance (discoloration), but in the UV section of the spectra, other bands appeared as a function of the ozonation time and disappeared for longer times, showing that the degradation is constituted by a degradation mechanism involving several steps, with the first step corresponding to discoloration. Table 1 presents the discoloration kinetics parameters resulting from the ozonation of the aqueous solutions of the dyes. Because textile-dying processes involve high concentrations of salts, particularly sodium chloride, ozone degradation was also studied in the presence of NaCl (50 g/L), and the results are shown in Table 1.

Analysis of Table 1 shows that the apparent rate constants in pure dye aqueous solutions are in the 0.1 min^−1^ range, which allows for more than 90% discoloration in less than 30 min. For MO, the obtained *k*_ap_ was 0.210 min^−1^, which is higher than values reported in the literature. For example, for an MO concentration of 500 mg/L in distilled water (pH = 9, ozone mass flux = 109 mg/h) the *k*_ap_ was 0.044 min^−1^ (at 20 °C) and 0.039 min^−1^ (at 30 °C) [39].

When NaCl is present, a marked increase in the apparent rate constant is observed. This increase may be due to generation of other radicals that result from the reaction of ozone and chloride [40], which contribute to the degradation of the dye structures. When chloride is present in the aqueous solution and ozone is added, a new AOP is generated that is efficient for dye degradation. The chemical reaction than initiates the chain reaction is expressed as follows [40,41]:

O_3_ + Cl^−^ → O_3_*^−^ + Cl* (13)

O_3_ + Cl* → O_2_ + ClO* (14)

Cl* + Cl^−^ → Cl_2_*^−^ (15)

These radicals will have an active role on dye degradation or, indirectly, by increasing the concentration of radical hydroxide.

### 3.2. Preliminary Analysis of the Catalytic Performance of the Cu-CD

A preliminary exploratory synthesis design was used to obtain the highest potential catalyst from a basic set of reagents. The three questions that this preliminary design answered were: (i) which carbon precursor should be used (L-cysteine, citrate or ascorbic acid); (ii) the necessity of PEG in the formulation; (iii) the necessity of a metal cation and which it should be (copper(II), zinc(II) or titanium(IV)). The nanocomposites obtained with these reagent combinations were used as catalyst in the ozone decomposition of MO, and the corresponding apparent constant rates were compared with those without catalysis, as presented in the previous section.

This exploratory analysis showed that without PEG, no catalysis was observed and that a significant increase on the apparent constant rate was observed when copper(II) and L-cysteine were used. In order to define an optimal initial set for the experimental factors to obtain the nanocomposite that shows higher catalytic properties a Plackett-Burman experimental design with two central experiments (a total of 10 experiments) was used (Section 2.2). The ANOVA of the results (Supplemental Information) shows that neither factor is statistically significant. However, PEG and the reaction time show some effect, i.e., the corresponding standard error is lower than the coefficient (Table S1, and the corresponding response surface (Figure S2 shows that a higher PEG volume and a lower reaction time result in Cu-CD with higher catalytic activity. The optimal synthesis conditions of Cu-CD are described in Section 2.2.

### 3.3. Characterization of the Cu-CD

The lyophilization of the homogeneous aqueous solution originated a gelatinous material. This material, which is highly water soluble, resulted from the caramelization of the PEG, and accordingly to the results of the preliminary exploratory analysis, it is crucial for the discoloration catalytic performance of the Cu-CD.

**Figure 2:**
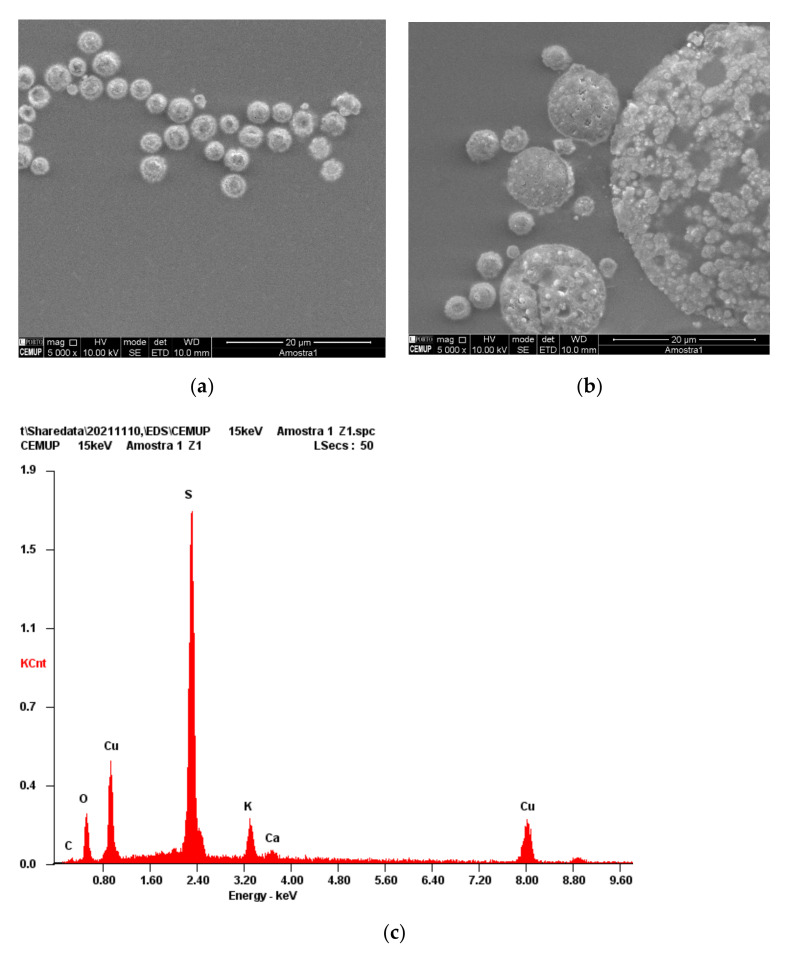
(**a**,**b**) SEM images of Cu-CD and (**c**) the corresponding EDX.

Figure 2a,b shows SEM images of the Cu-CD nanoparticles. The analysis of these images shows that the material is highly agglomerated into spherical structures. These structures are highly heterogeneous and composed of smaller identical spherical structures. EDS analysis confirms that the copper dopes the nanomaterial, as well as the presence of S, C and O from the organic precursors (Figure 2c). The relatively low intensity of the peak due to carbon and oxygen is due to the relatively low sensitivity of EDS towards lighter elements and to the imaging scaling effect due to the existence of heavier elements in relatively large amounts, such as sulfur. This result confirms that the designed surface composition of the Cu-CD was obtained, i.e., the copper(II) ions and thiol functionalities constitute the surface reactive sites.

**Figure 3:**
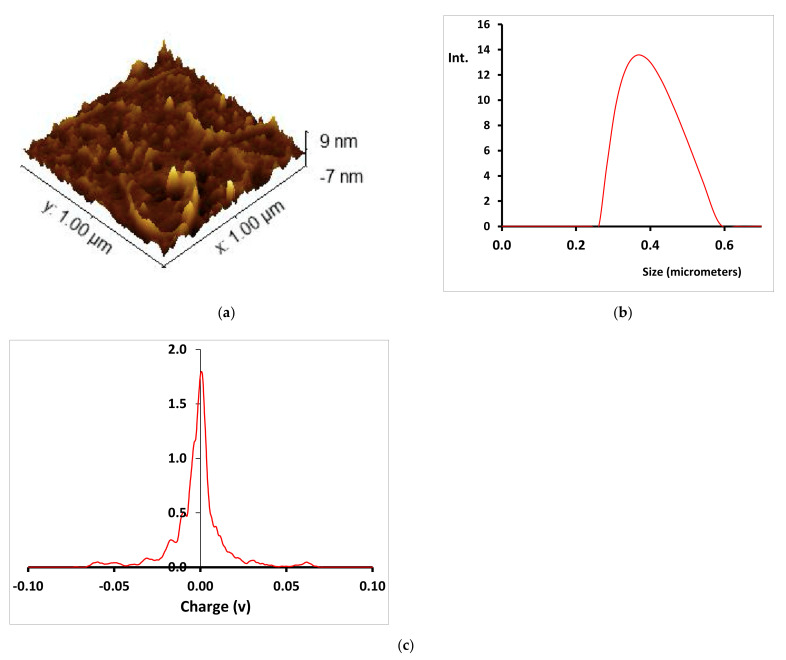
(**a**) AFM image, (**b**) DLS spectra and (**c**) Zeta potential of Cu-CD.

Figure 3a shows the AFM images of the Cu-CD. The analysis of the AFM results shows that the Cu-CD had an average size of 4.0 ± 1.5 nm, which is in agreement with the size of other uniform spherical-morphology copper(II)-based CDs of 4.84 nm [29]. In aqueous solution, the nanomaterial becomes highly agglomerated and hydrated, as shown by the DLS results in Figure 3b, with an average hydrodynamic size of about 390 nm. This result is in agreement with the existence of a highly hydrated caramelized capping in the Cu-CD that promotes the association or agglomeration of individual nanoparticles. Figure 3c shows that the mean zeta potential is −3.7 mV, with a charge distribution ranging from slightly positively charged to slightly negatively charged nanoparticles.

**Figure 4:**
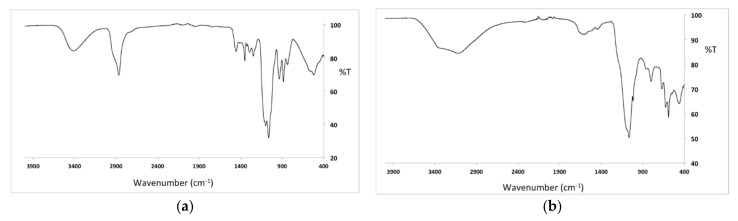
FTIR spectrum of Cu-CD (**a**) and CuCys-CD (**b**).

The infrared spectrum of Cu-CD, shown in Figure 4a, is similar to the spectrum of CuCys-CD (Figure 4b), with some more bands due to the PEG coating the nanoparticles. Besides the bands due to PEG, the IR spectra of both samples are constituted by the following main bands [42,43]: C–O stretching at 1064 cm^−1^, C=C stretching at 1643 cm^−1^, C−H stretching mode at 3142 cm^−1^ and O-H stretching at 3378 cm^−1^. The main bands due to the PEG are [43]: C–O, C–C stretching and CH_2_ rocking at 845 cm^−1^; CH_2_ rocking and CH_2_ twisting at 940 cm^−1^; C–O, C-C stretching and CH_2_ rocking at 1066 cm^−1^; C–O and C–C stretching at 1106 cm^−1^; C–O stretching and CH_2_ rocking at 1145 cm^−1^; CH_2_ twisting at 1254 cm^−1^; CH_2_ wagging at 1352 cm^−1^; CH_2_ scissoring at 1461 cm^−1^; C-H stretching at 2874 cm^−1^; and O-H stretching at 3441 cm^−1^.

**Figure 5:**
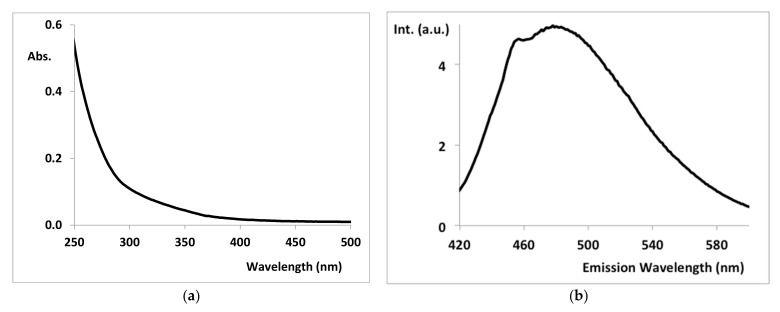
(**a**) UV-Vis and (**b**) fluorescence emission (ex. 350 nm) spectra of Cu-CD.

Figure 5 shows the UV-Vis (Figure 5a) and fluorescence emission at the maximum of the excitation (350 nm) (Figure 5b) spectra. The UV-Vis spectrum (Figure 5a) corresponds to a typical CD, with a strong absorption in the ultraviolet, tailing into the visible region [22]. Although not clear, there is a slight shoulder in the UV-Vis spectrum at about 350 nm (the maximum of the fluorescence excitation), which may be attributed to electronic transitions from C–O or C=O bonds to π* orbitals [19]. The fluorescence emission spectrum has two overlapped bands with maxima at 454 and 477 nm, which is responsible for a typical blue emission of CD [16,17,19].

Although the objective was not to synthesize CD with high quantum yield (QY) but with ozonation catalytic capabilities, the QY of the synthesized Cu-CD was quite high, namely: raw Cu-CD, 31%; lyophilized Cu-CD (solution with a concentration of 0.20 mg/L), 21%. This relatively high value confirms that the fluorescent nanomaterial, the CD, became coated with PEG during the one-pot button-up hydrothermal synthesis, resulting in relatively high QY values [31,32,33].

### 3.4. Characterization of the CuCys-CD

Taking into consideration that Cu-CD is obtained after lyophilization as a gelatinous material some, characterization could not be performed, namely XPS and XRD analysis. For this reason, a carbon dot was prepared using the same synthesis methodology as that for Cu-CD but without PEG, obtaining a powder sample named CuCys-CD. As discussed above, CuCys-CDs have no catalysis capabilities with respect to the ozone AOP, but their analysis will elucidate, indirectly, more features of the Cu-CD.

The Zeta potential of CuCys-CD (Figure S3 of the Supplemental Information) shows that the mean zeta potential is 6.7 mV, with the majority of the charge distribution in the positively charged side. This result shows that the coating of CuCys-CD with PEG, resulting in the Cu-CD, neutralized the positive charge of these nanoparticles.

**Figure 6:**
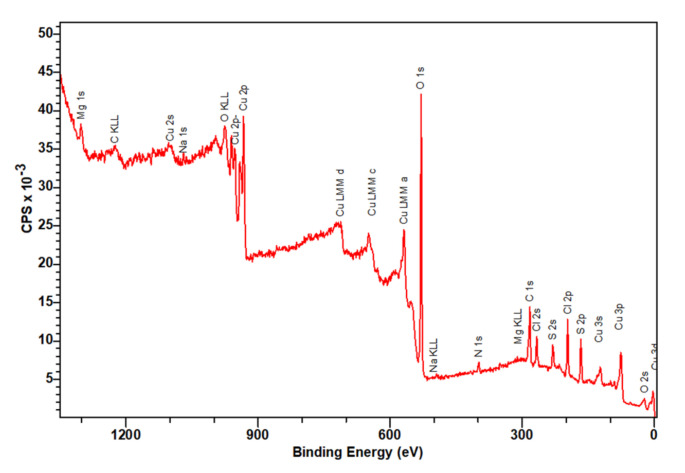
XPS survey scan of CuCys-CD.

**Figure 7:**
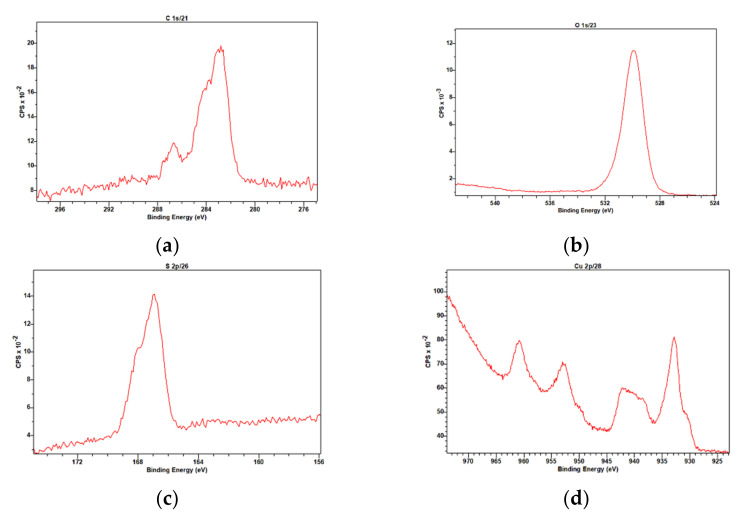
High-resolution XPS C1s (**a**), O1s (**b**), S2p (**c**) and Cu2p (**d**) spectra of CuCys-CD.

Figure 6 shows the XPS survey scan of CuCys-CD. As expected, the XPS shows that the elemental analysis of the surface of the nanomaterial is constituted by the elements carbon, oxygen, sulphur and copper. The binding energy of the main band of carbon (C1s) is 283 eV, with a shoulder at 284 eV and a smaller band at 287 eV (Figure 7a). The bands at about 284 eV may be assigned to an sp2 carbon in graphite, and at 287 eV, to carbon bound to oxygen in C-O-C structures [44]. The binding energy of the oxygen (O1s) is 530 eV (Figure 7b). This large band may be due to metal oxides and organic C-O [44]. The binding energy of the sulphur (S2p) is 167 eV, with a shoulder at 168 eV (Figure 7c) [44]. These bands may be assigned to Na_2_(SO_3_)_2_ or metal sulphates [44]. The binding energy of the main band of copper (Cu2p) is 933 eV, which may correspond to metallic copper, copper(I) and/or copper(II) (Figure 7d). However, analysis of the satellite features (bands at 941, 953 and 961 eV) provides further information about the oxidation state of copper in the nanomaterial, and the unequivocal oxidation state of copper is (II) (Figure 7d) [44].

**Figure 8:**
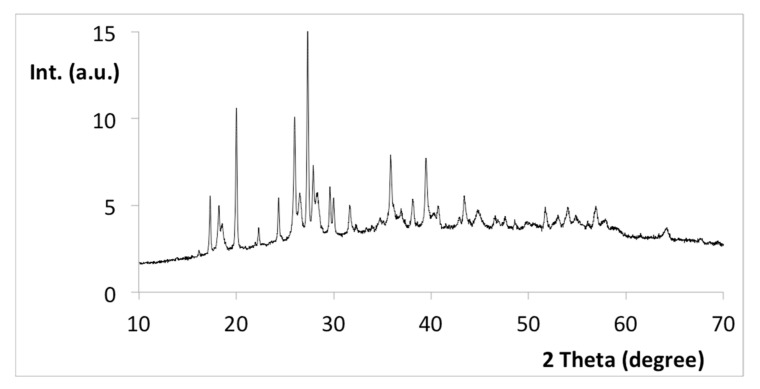
XRD spectrum of CuCys-CD.

The high-resolution XRD spectrum of CuCys-CD (Figure 8) shows a main set of bands between 26 and 28 degrees, which correspond to the reflection in the (002) plane of aromatic layers, as well as a week peak at about 43 degrees, which corresponds to the reflection in the (100) plane of aromatic layers [45]. This result suggests that the carbon dots synthesised in this work have a graphitic core. Other main peaks are observed in the ranges between 17 and 20 degrees and between 35 and 41 degrees, which may be due to copper oxide nanoparticles [46]. This result is in agreement with the XPS information about the existence of copper(II) in the prepared carbon dots.

### 3.5. Ozone AOP in the Presence of Cu-CD

The effect of the addition of Cu-CD in the ozonation degradation kinetics of the four dyes is shown in Table 2. Comparative analysis of the apparent rate constants with the catalyzer (Table 2) with those without a catalyst (Table 1) shows that the pure dye discoloration rate is markedly increased when Cu-CD is present, ranging from 167 to 653%. An increase on the degradation of the dyes is also observed for the salt-containing solutions, although with a relatively low magnitude, ranging from 4 to 80%. This difference is due to the fact that in the salt solutions, the number of reactive species present in solution is much higher than in pure water, and consequently, the increment due to the Cu-CD is relatively smaller.

The presence of Cu-CD provoked the following increases in the apparent first-order rate constant: MO, 429% in the constant rate (*k*_ap_^c^ = 1.184 ± 0.003 min^−1^); O-II, 653% in the constant rate (*k*_ap_^c^ = 1.002 ± 0.036 min^−1^); RB-5, 301% in the constant rate (*k*_ap_^c^ = 0.709 ± 0.029 min^−1^); RBB-R, 167% in the constant rate (*k*_ap_^c^ = 0.230 ± 0.004 min^−1^). These discoloration rate values are much higher than the majority of results reported in the literature for the same dyes using other AOPs [39,41,47,48,49,50,51,52,53,54,55,56,57,58].

A catalytic ozonation process of Ni-based layered double-hydroxide (Ni-LDHs) nanomaterials was used for MO degradation, with a *k*_ap_^c^ of 0.053 min^−1^ (20 °C) and 0.050 min^−1^ (30 °C) [39]. Other nanoparticles used in the MO degradation processes resulted in *k*_ap_^c^ of 0.559 min^−1^ (Fe^0^ nanoparticles), 2.1 × 10^−3^ min^−1^ (nanoporous Au), 0.0515 min^−1^ (TiO_2_ nanotubes arrays), 0.1025 min^−1^ (Fe^0^-UV-H_2_O_2_) and 2.444 min^−1^ (hollow Co nanoparticles) [41]. A photocatalytic reactor using an ozone-generating mercury vapor lamp was used for MO degradation, and the *k*_ap_^c^ ranged between 0.0309 min^−1^ (VUV + O_3_) and 0.0630 min^−1^ (VUV + TiO_2_ + O_3_) [47]. A photocatalytic discoloration of MO by δ-Bi_2_O_3_ thin films obtained *k*_ap_^c^ ranging from 0.0070 to 0.0500 min^−1^ [48]. The discoloration of MO and O-II by the δ-MnO_2_/formic acid system obtained *k*_ap_^c^ = 0.2729 min^−1^ (MO) and *k*_ap_^c^ = 0.0852 min^−1^ (O-II) [49]. Heterogeneous Fenton and Fenton-like reactions using alginate-Fe^2+^/Fe^3+^ films as catalysts for MO degradation obtained *k*_ap_^c^ ranging from 0.008 to 0.117 min^−1^ [50].

Four materials containing Fe were used as photochemical heterogeneous catalysts for the discoloration and mineralization of O-II in the presence of H_2_O_2_ and UVC (pH values of 3 and 6), and the *k*_ap_^c^ changed from 0.050 to 0.193 min^−1^ [51]. A bentonite clay-based Fe nanocomposite was used as a catalyst in a photo-Fenton discoloration and mineralization of azo-dye O-II, and the *k*_ap_^c^ (at three temperatures of 30, 46 and 60 °C) changed from 0.1308 to 0.2686 min^−1^ [52]. A Fe^3^-doped TiO_2_ and a bentonite clay-based Fe heterogeneous photo-Fenton nanocatalyst allowed for the discoloration and mineralization of O-II in about 80 min [53].

A zeolite (MNZ) was impregnated with different amounts of Fe in order to study the photo-Fenton degradation of RB5, and the *k*_ap_^c^ changed from 0.0005 to 0.0447 min^−1^ [54]. A TiO_2_–zeolite nanocomposite designed for the photocatalysis of RB-5 resulted in the complete degradation of the dye in 95 min (*k*_ap_^c^ = 0.0102 min^−1^) [55].

A study on the RBB-R biodegradation using the fungus T. hirsuta D7 immobilized in LECA obtained a *k*_ap_ = 0.0022 min^−1^ [56]. The RBB-R discoloration was mediated by the fungi *T. citrinoviride*, *T. koningiopsis* and *Pestalotiopsis sp*. strains, and *k*_ap_^c^ changed from 0.1077 to 0.1914 min^−1^ [57]. Palygorskite was modified with TiO_2_ to produce a photocatalyst for the degradation of RBB-R, with *k*_ap_^c^ varying from 0.0610 × 10^−2^ to 0.0687 × 10^−2^ min^−1^ [58].

### 3.6. Real Textile Effluent Analysis

In order to assess whether the Cu-CD has potential for the catalysis of the degradation of real textile effluent, a sample from a textile dying and printing industry was studied (Figure S4 from Supplemental Information. The UV-Vis spectrum of the raw effluent is characterized by a visible band at about 600 nm and a UV shoulder at about 365 nm. The absorbance of the spectrum is relatively small due to the dilution of the rejected dyes with the huge amount of water used in all textile industrial processes [59].

**Figure 9:**
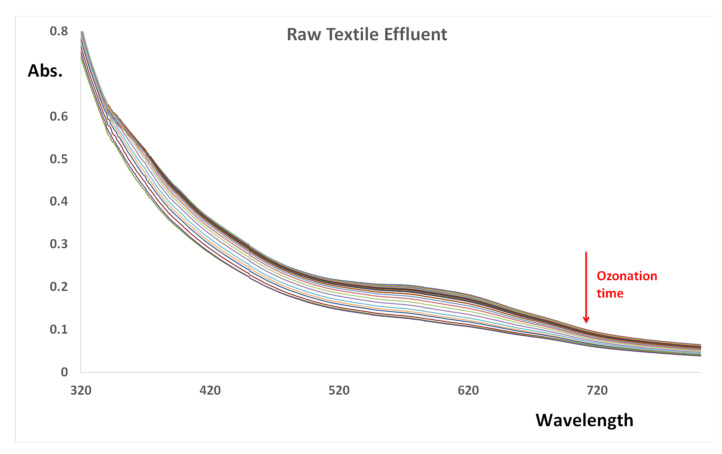
UV-Vis spectra of a real textile dying and printing industry as a function of ozonation time.

As shown in Figure 9 (and in Figure S4 from Supplemental Information) the ozonation of the raw effluent provoked an overall decrease in the ultraviolet and visible absorbance. Ozone treatment of the raw effluent allows for a dye removal of 48% after 60 min, with a *k*_ap_ = 0.0097 min^−1^. The presence of Cu-CD resulted in a %Inc = 24%, with a *k*_ap_^c^ = 0.012 ± 0.001 min^−1^ and a %DR = 41.0%. Besides the observed color reduction in the effluent (image S4 in SI), the COD and turbidimetry also decreased relative to the original values (Section 2.1) to 310 ± 15 mg O_2_/L and 44 ± 7 NTU, respectively.

Apparently, the ozonation of the raw effluent had a markedly worse performance than that observed with the pure aqueous dye solutions. This result may be due to the following: (i) type of predominant dye in the sample of real effluent, which seems to be blue dyes, which, like anthraquinone RBB-R, have relatively low reactivity towards ozone; (ii) many other substances and microorganisms that are present in the sample compete with the dyes towards the reactive species resulting from ozone, reducing its efficiency; and (iii) the main factor may be the presence of ozone decomposition inhibitors in the wastewater.

Among the inhibitors, the carbonates, which are present in the effluent at concentrations higher than 356 mg/L, are the most well-known [60,61]. The origin of large amounts of carbonates in textile wastewater are two-fold [61]: (i) dyeing processes, for example, involving reactive dyes, require alkaline aqueous medium, which is achieved by using large amounts of carbonate; (ii) the first wastewater treatment is pH neutralization to near-neutral values, and this is achieved using carbon dioxide, for example, by bubbling the flue gas of the fossil fuel burning process, which is sequestered in the water as carbonate. Carbonate acts as a scavenger of the hydroxyl radical (*OH) and of the ozone anion, O_3_*^−^ [60]. As presented in the introduction above, these two radical species are the propagators of the ozone decomposition, and the presence of carbonate contributes to ozone stabilization (inhibitor of ozone decomposition). Because the decomposition of the dyes occurs mainly via the radical species produced during ozone decomposition, the presence of carbonate reduces the efficiency of the discoloration of the dye solutions [60]. The chemical equations that correspond to these carbonates (carbonate and bicarbonate anions) reactions are:

CO_3_^2−^ + *OH → CO_3_*^−^ + HO^−^ (16)

CO_3_^2−^ + *OH → CO_3_*^−^ + HO^−^ (17)

CO_3_*^−^ + O_3_*− → CO_3_^2−^ + O_3_ (18)

The presence of high amounts of carbonates in textile wastewater constitutes a critical limitation to the use of ozone in its discoloration, the water must be preprocessed to some extents, for example, by the addition of calcium cations to precipitate the carbonates.

### 3.7. Mechanism of Ozone plus Cu-CD

The catalytic effect of Cu-CDs may result from the following process that occur on their surface:

(i). Taking into consideration the high specific surface area of the nanocomposite and that the carbon-based core is passivated by a hydrated soft caramelized PEG coating, the dyes can become adsorbed on the surface.
(ii). Secondly, on the same surface, ozone is catalytically transformed into hydroxyl radicals due to the existence of copper and sulfur atoms.
(iii). Thirdly, the adsorbed dyes react with the hydroxyl radicals being degraded.

A scheme of this hypothetical mechanism is shown in Figure S5 of the Supplemental Information. One of the protagonists of this dye degradation process is PEG, which we observed to be the most significant and fundamental factor in the catalysis features of Cu-CD. Apparently, PEG creates a coating on the nanoparticle that concentrates the reagents necessary for the degradation to occur.

To confirm that the hydroxyl radicals are involved in the Cu-CD-based catalytic mechanism, hydroxyl radical scavenging experiments were carried out using isopropyl alcohol, and the obtained results were compared with the absence of any scavenger [62]. The addition of isopropyl alcohol significantly inhibits MO degradation, i.e., the *k*_ap_ reduces by about 30% (1.184 ± 0.003 and 0.83 ± 0.01 min^−1^ without and with isopropyl alcohol, respectively), clearly indicating that the hydroxyl radical is the main oxidative active species involved in dye degradation when Cu-CDs are present.

## 4. Conclusions

A copper(II)-doped CD produced by a one-pot hydrothermal methodology using L-cysteine and PEG as precursors was obtained that significantly catalyzes the ozonation of aqueous solutions of dyes at pH = 7.0 and T = 25 °C. The sample of Cu-CD provoked an increase in the discoloration rate, allowing for 90% efficiency in about 6 min for the azo dyes (MO, O-II and RB-5) and 30 min for the anthraquinone dye (RBB-R).

It was observed that NaCl, a very common substance in textile effluents, contributes positively to dye degradation in the ozone AOP. Indeed, the presence of chloride releases further radical species that degrade dyes.

Although the Cu-CD-based mechanism catalyzes the ozonation of real textile wastewater, the efficiency of this methodology in the treatment of raw effluents is not satisfactory. Indeed, catalytic ozonation is inhibited by a ubiquitous substance in textile sewers, carbonates, which limits the success of its application. Consequently, wastewater must be preprocessed in order to eliminate/separate the main inhibitors. Following this pretreatment, in this work, an almost complete discoloration was obtained in a minute time scale.

## Figures and Tables

**Table 1:** Apparent rate constant and percentage of dye removal of the ozonation AOP without and with 50 g NaCl/L (the standard deviations of at least three independent repetitions are shown). The reaction time necessary to achieve the percentage of dye removal is shown in parentheses. Fitting of the integrated first-order kinetics resulted in R^2^ > 0.98.

Dye	*k*_ap_ (min^−1^)	%DR
MO	0.210 ± 0.013	99.4 ± 0.3 (30 min)
MO + NACL	0.631 ± 0.046	99.4 ± 0.1 (10 min)
O-II	0.133 ± 0.021	97.9 ± 0.7 (30 min)
O-II + NACL	0.642 ± 0.034	99.0 ± 0.4 (10 min)
RB-5	0.177 ± 0.031	98.4 ± 0.5 (30 min)
RB-5 + NACL	1.013 ± 0.010	98.1 ± 0.1 (4 min)
RBB-R	0.086 ± 0.009	91.4 ± 0.9 (30 min)
RBB-R + NACL	0.123 ± 0.003	96.7 ± 0.5 (30 min)

**Table 2:** Apparent constant rate and percentage of dye removal of the ozonation AOP in the presence of Cu-CD (the standard deviation of at least three independent repetitions is shown). The number of times the k_ap_ increased relative to the uncatalyzed reaction (Table 1) and the reaction time necessary to achieve the percentage of dye removal is shown in parentheses. Fitting of the integrated first-order kinetics resulted in R^2^ > 0.98.

Dye	*k*_ap_^c^ (min^−1^)	%Inc	%DR
MO + CU-CD	1.184 ± 0.003	429	99.8 ± 0.2 (6 min)
MO + NACL + CU-CD	0.705 ± 0.006	12	99.1 ± 0.1 (10 min)
O-II + CU-CD	1.002 ± 0.036	653	99.3 ± 0.2 (6 min)
O-II + NACL + CU-CD	0.971 ± 0.009	51	99.7 ± 0.2 (10 min)
RB-5 + CU-CD	0.709 ± 0.029	301	80.1 ± 1.6 (6 min)
RB-5 + NACL + CU-CD	1.057 ± 0.047	4	86.0 ± 3.1 (6 min)
RBB-R + CU-CD	0.230 ± 0.004	167	99.1 ± 0.1 (30 min)
RBB-R + NACL + CU-CD	0.221 ± 0.004	80	98.7 ± 0.2 (30 min)

## Data Availability

Not applicable.

## Supplementary Materials

The following supporting information can be downloaded at: https://www.mdpi.com/article/10.3390/nano12071211/s1, Figure S1 - Spectra of dyes as function of the reaction time: (a) Methyl Orange; (b) Reactive Black 5; (c) Orange II; and (d) Remazol Brilliant Blue R, Figure S2 – Response surface of the *k*_ap_ as function of the factors PEG volume (mL) and time (hours), Table S1 – ANOVA of the Plackett-Burman experimental design results, Figure S3. Zeta-potential of the CuCys-CD, Figure S4. Real textile effluent before and after ozonation, and Figure S5. Scheme of the catalytic mechanism of Cu-CD.
