# Supplementary material for: Copper(II)-Doped Carbon Dots as Catalyst for Ozone Degradation of Textile Dyes
Source: Nanomaterials (Basel). 2022 Apr 4;12(7):1211. doi: 10.3390/nano12071211 (PMC9003027; doi:10.3390/nano12071211)
Supplement: Supplementary file 1 [file nanomaterials-12-01211-s001.zip › nanomaterials-1662433-supplementary.pdf]

nanomaterials

MDPI

Supplemental Information

Copper(II)-Doped Carbon Dots as Catalyst for Ozone Degradation of Textile Dyes

Rita M. F. Cardoso, Inês M. F. Cardoso, Luís Pinto da Silva and Joaquim C. G. Esteves da Silva *

Chemistry Research Unit (CIQUP), Institute of Molecular Sciences (IMS)—DGAOT, Faculty of Sciences of University of Porto (FCUP), Rua do Campo Alegre 697, 4169-007 Porto, Portugal; up201704723@edu.fc.up.pt (R.M.F.C.); up201704720@edu.fc.up.pt (I.M.F.C.); luis.silva@fc.up.pt (L.P.d.S.)
* Correspondence: jcsilva@fc.up.pt; Tel.: +351-220402569

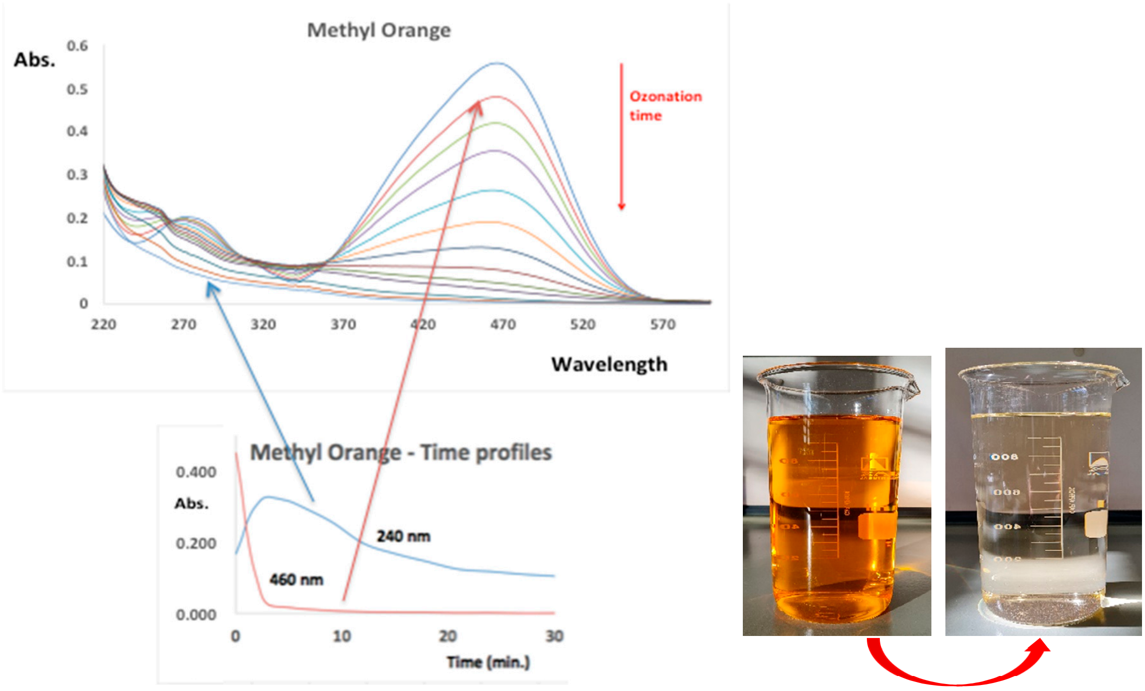

a.

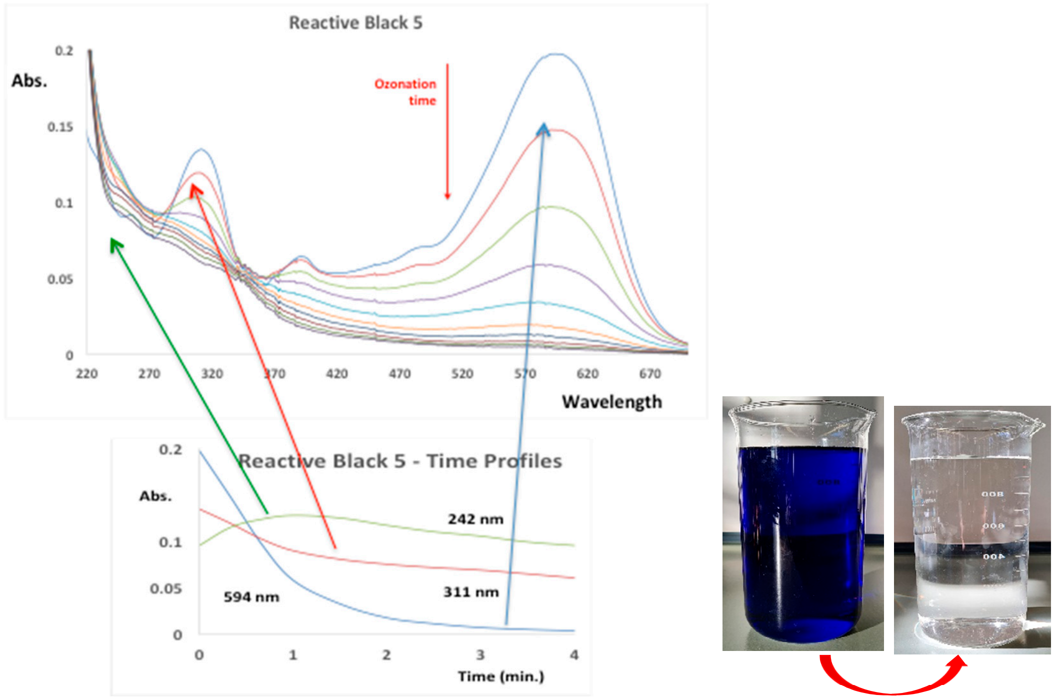
Reactive Black 5
Abs.
0.2
0.15
0.1
0.05
0
220
270
320
370
420
470
520
570
620
670
Wavelength
Ozonation time
Reactive Black 5 - Time Profiles
Abs.
0.2
0.1
0.05
0
242 nm
311 nm
594 nm
0
1
2
3
4
Time (min.)

b.

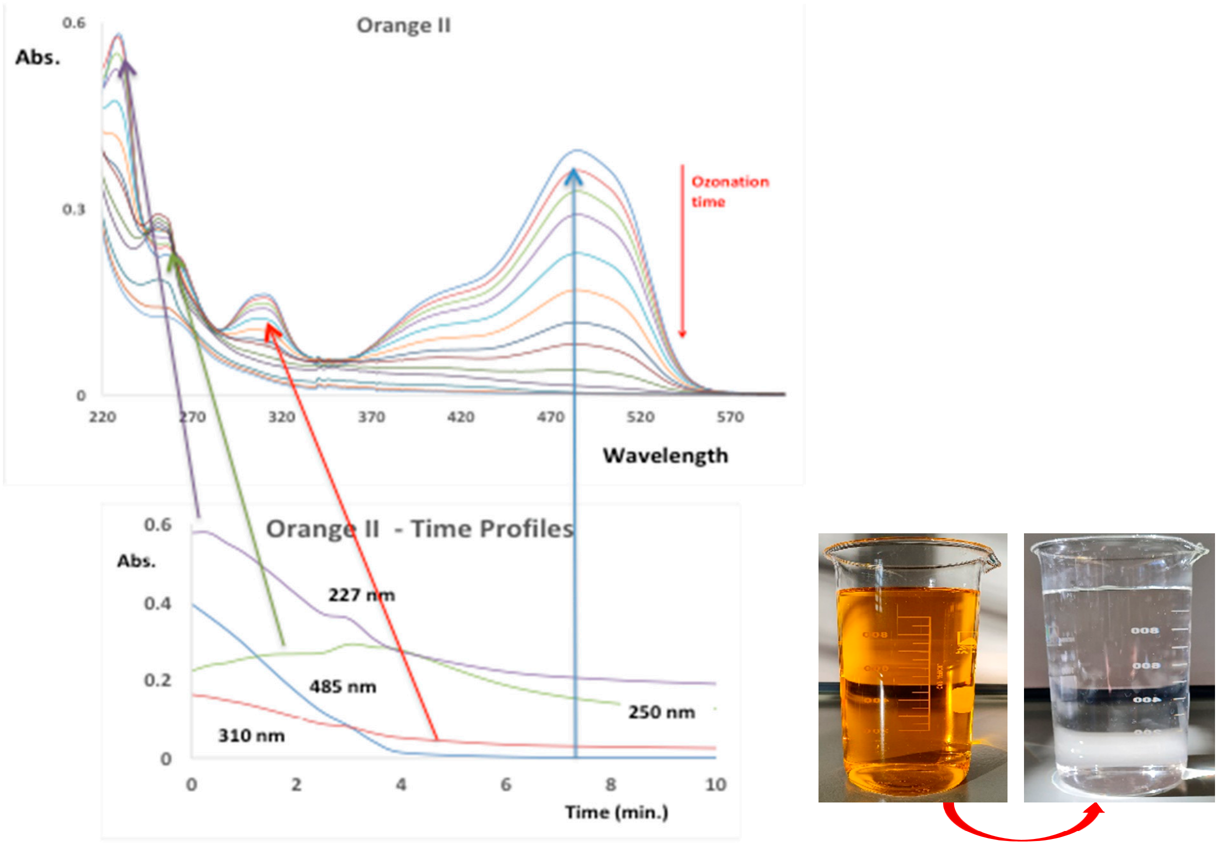
Orange II
Abs.
0.6
0.3
0
220
270
320
370
420
470
520
570
Wavelength
Ozonation time
Orange II - Time Profiles
Abs.
0.6
0.4
0.2
0
227 nm
485 nm
250 nm
310 nm
0
2
4
6
8
10
Time (min.)

c.

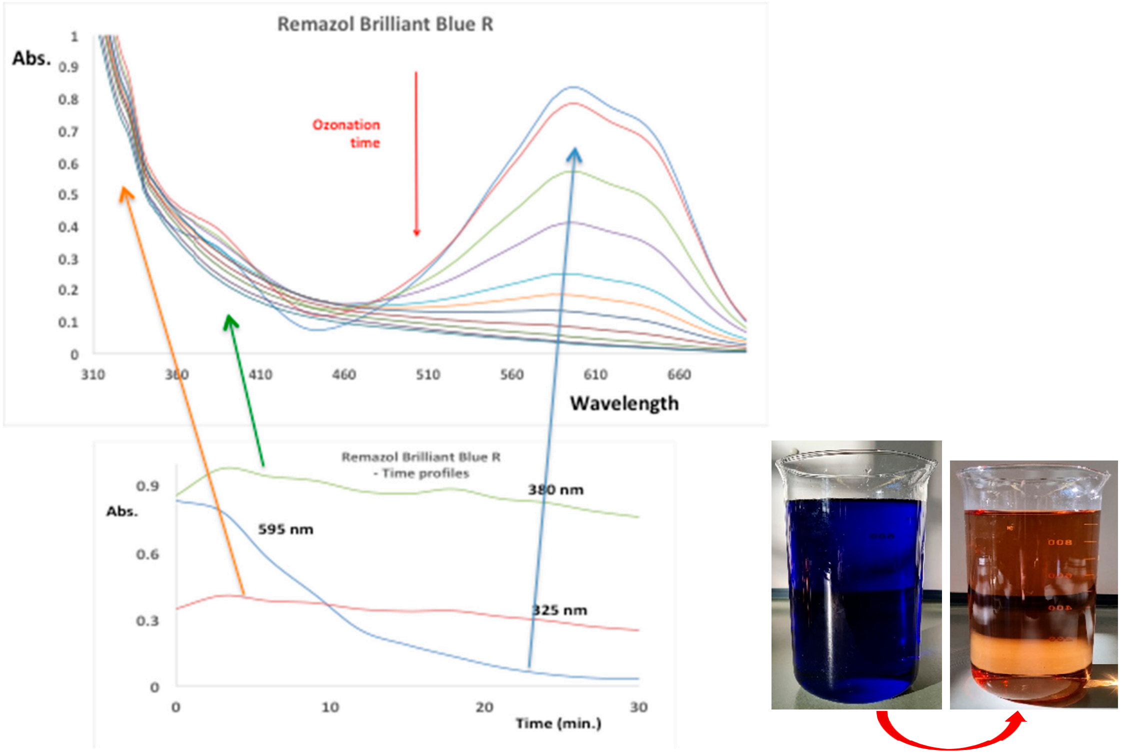

d.

Figure S1. - Spectra of dyes as function of the reaction time: (**a**) Methyl Orange; (**b**) Reactive Black 5; (**c**) Orange II; and (**d**) Remazol Brilliant Blue R.

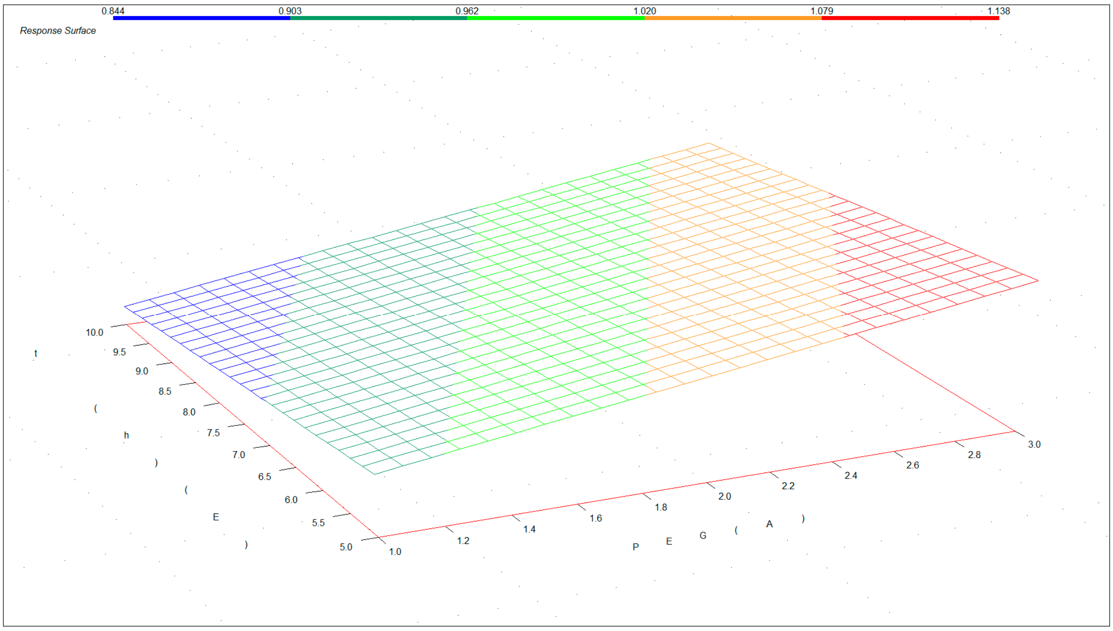

Figure S2. – Response surface of the k_{ap} as function of the factors PEG volume (mL) and time (hours).

Table S1. – ANOVA of the Plackett-Burman experimental design results [multiple correlation: 0.755 (cal); R-Squared: 0.579 (cal); Uncentered B0-coeficient: 1.702].

	SS	DF	MS	F-ratio	p-value	B-coeff.	STDerr
**Summary**							
**Model**	0.119	5	0.02388	1.062	0.4903		
**Error**	0.08990	4	0.02247				
**Adjusted Total**	0.209	9	0.02325				
**Variable**							
**Intercept**	9.822	1	9.822	437.042	0.0000	0.991	0.04741
**PEG (A)**	0.07685	1	0.07685	3.419	0.1381	0.009801	0.05300
**Cysteine (B)**	0.004045	1	0.004045	0.180	0.6932	-0.150	0.353
**Copper (C)**	0.01127	1	0.01127	0.502	0.5179	-0.150	0.212
**T (ºC) (D)**	0.008263	1	0.008263	0.368	0.5770	-0.00321	0.00530
**t (h) (E)**	0.0189	1	0.0189	0.844	0.4103	-0.01947	0.02120
**Lack of Fit**							
**Lack of Fit**	0.08872	3	0.02957	25.145	0.1453		
**Pure Error**	0.001176	1	0.001176				
**Total Error**	0.08990	4	0.02247				

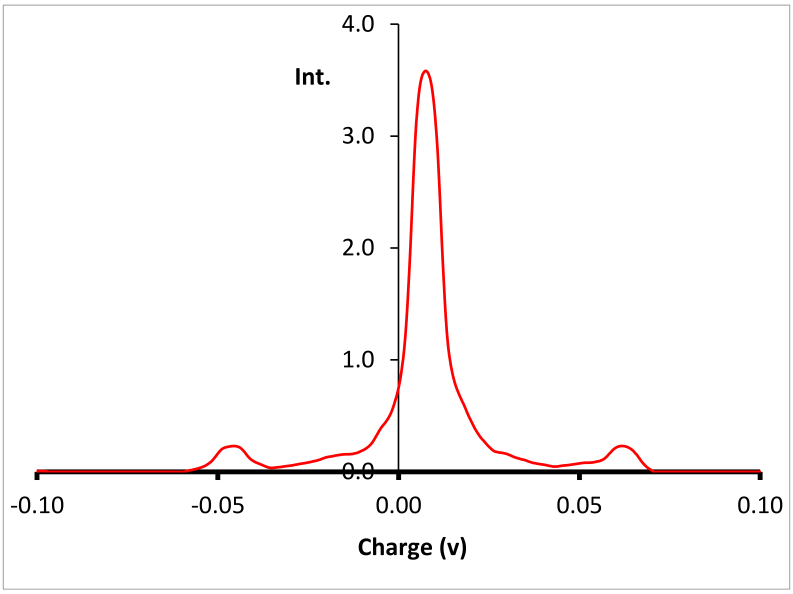

Figure S3. Zeta-potential of the CuCys-CD.

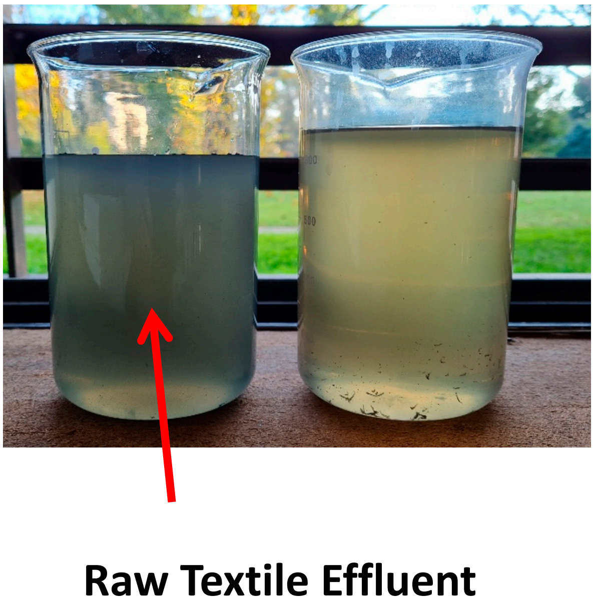

Figure S4. Real textile effluent before and after ozonation.

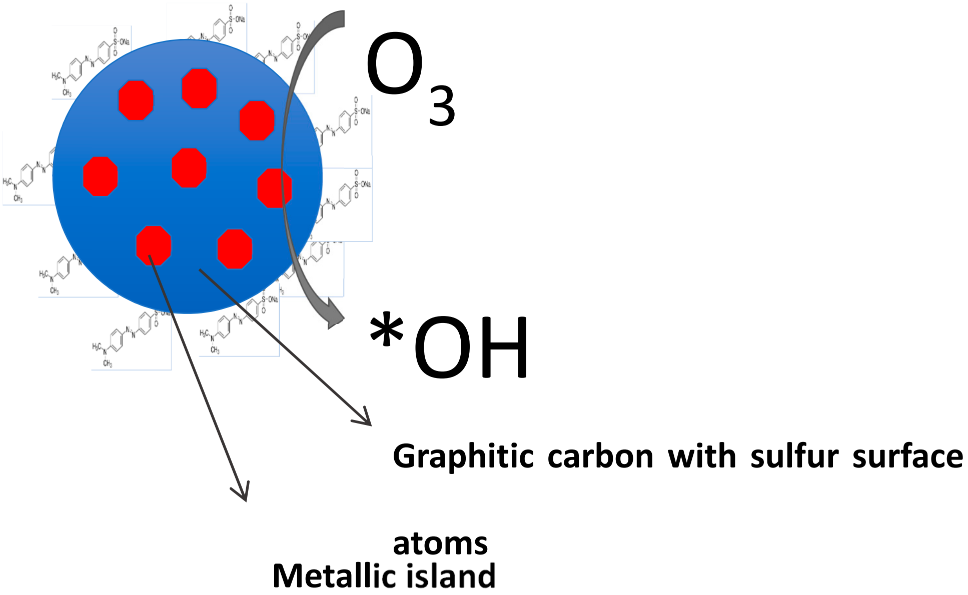

Figure S5. Scheme of the catalytic mechanism of Cu-CD.
